# Ab-initio investigation of preferential triangular self-formation of oxide heterostructures of monolayer $\hbox {WSe}_{2}$

**DOI:** 10.1038/s41598-020-78812-2

**Published:** 2020-12-10

**Authors:** Soumya Ranjan Das, Katsunori Wakabayashi, Kazuhito Tsukagoshi, Sudipta Dutta

**Affiliations:** 1 Department of Physics, Indian Institute of Science Education and Research (IISER) Tirupati, Tirupati, Andhra Pradesh 517507 India; 2 Department of Nanotechnology for Sustainable Energy, School of Science and Technology, Kwansei Gakuin University, Gakuen 2-1, Sanda, Hyogo 669-1337 Japan; 3 WPI Center for Materials Nanoarchitectonics (WPI-MANA), National Institute for Materials Science (NIMS), Tsukuba, 305-0044 Japan

**Keywords:** Two-dimensional materials, Electronic properties and materials

## Abstract

Triangular growth patterns of pristine two-dimensional (2D) transition metal dichalcogenides (TMDs) are ubiquitous in experiments. Here, we use first-principles calculations to investigate the growth of triangular shaped oxide islands upon layer-by-layer controlled oxidation in monolayer and few-layer $\hbox {WSe}_{2}$ systems. Pristine 2D TMDs with a trigonal prismatic geometry prefer the triangular growth morphology due to structural stability arising from the edge chalcogen atoms along its three sides. Our ab-initio energetics and thermodynamic study show that, since the Se atoms are more susceptible to oxygen replacement, the preferential oxidation happens along the Se zigzag lines, producing triangular islands of transition metal oxides. The thermodynamic stability arising from the preferential triangular self-formation of TMD based oxide heterostructures and their electronic properties opens a new avenue for their exploration in advanced electronic and optoelectronic devices.

## Introduction

The successful synthesis of graphene^1,2^ has led to an impressive surge in the exploration of atomically thin layered materials. Due to their reduced degrees of freedom, the quantum mechanical effects become significant, giving rise to fascinating electronic, mechanical, and optical properties otherwise absent in bulk materials^3,4^. However, the absence of a band gap in graphene presents a serious impediment in semiconductor electronics applications. This has tempted researchers to explore other low dimensional materials with varying functionalities^5–10^. Among them, 2D semiconducting TMDs, like $\hbox {WSe}_{2}$ and $\hbox {MoS}_{2}$, have attracted renewed interest because of its sizeable direct band gap and high carrier mobility at room temperature, which has interesting electronic, optoelectronic and valleytronic properties^11–16^. The high on-off ratio and intrinsic voltage gain of semiconducting 2D TMDs have enabled them to be used as atomically thin transistors^12,17–21^, photodetectors^22–24^ and light-emitting diodes^25–27^ with performance comparable to the well-known silicon devices.

2D TMDs can be prepared by various methods, like vapor-phase chemical reactions, wet-chemical synthesis and liquid exfoliation^13,28^. Apart from the edges or defect sites, which are chemically active, pristine layered dichalcogenides are mostly passive due to the absence of any dangling bonds^29^. Despite the variety of properties, all monolayer pristine TMD systems with trigonal prismatic geometry are obtained as triangular nanoflakes on synthesis^11^. Experimental and theoretical studies on the growth mechanism of monolayer TMD nanoclusters suggest that the triangular geometry with all chalcogen atom edges are energetically most favorable^30–33^. Chalcogen terminated triangular nanodots of TMDs exhibit many interesting physical and chemical properties, like strong spin anisotropy near the Fermi energy with collective spin states at the edges that can be utilized in efficient spin-filtering devices^34^.

Our recent experimental and theoretical work^35,36^ in monolayer and few-layer $\hbox {WSe}_{2}$ systems has further shown that, in the presence of an ozone environment, the surface Se atoms are preferentially replaced by O atoms, forming triangular oxide patches on pristine TMDs. This layer-selective oxidation of $\hbox {WSe}_{2}$ films has also been realized using laser heating^37^, ambient air^38^ and oxygen plasma doping, which is similar to conventional semiconductors^39^. The triangular island formation has also been reported in other studies^40^. Due to the temperature-dependent oxidation process, the triangular oxide patches increase in size, keeping their shape intact, until the full surface gets oxidized. The next layer of underlying pristine TMD starts getting oxidized only at elevated temperatures. This atomically thin TMD based oxide-semiconductor heterostructure created by layer-by-layer oxidation has interesting tunable electronic properties^36^. Moreover, charge transfer to the surface oxide layer makes them hole-doped^35^. Hence, these heterostructures can be used as field-effect transistors^41^ and photogating devices^42^.

Motivated by the enormous potential of these TMD based oxide heterostructures in electronic and optoelectronic device applications and their unique electronic properties, here we theoretically investigate these systems. We take a cue from the preferential self-formation of triangular nanoflakes of pristine TMDs and explore the underlying reason for similar triangular growth morphology of oxide islands on such pristine TMD systems. We have chosen our system to be $\hbox {WSe}_{2}$. However, our observations are expected to remain unchanged for any semiconducting 2D TMD system with trigonal prismatic geometry, as observed in our previous study^36^. We employ ab-initio techniques to capture the quantum mechanical effect in these reduced dimensions and systematically present our results in the following sections.

## Computational method

For structural relaxation and to study the electronic properties, we use first-principles calculations based on density functional theory (DFT) as implemented in the Vienna ab-initio simulations package (VASP)^43^. The calculations are performed at the level of the generalized gradient approximation (GGA) with the Perdew-Burke-Ernzerhof (PBE) exchange and correlation functional^44^. The electronic wave-functions are expanded using a plane-wave basis set with a cutoff energy of 520 eV. The 2D periodic tungsten selenide-oxide heterostructures are represented in a $4 \times 5$ super cell. A vacuum space of 15 Å is applied along the non-periodic direction to avoid any interaction within adjacent unit cells. For geometric relaxation and subsequent electronic property calculations, we consider Brillouin zone sampling over a $4 \times 4 \times 1$ and $16 \times 16 \times 1$ $\Gamma$-centered Monkhorst-Pack k-mesh grid, respectively. All structures are relaxed along with the lattice vectors until the force on each atom reaches 0.01 eV/Å. The convergence criterion for electronic minimization is set to $10^{-5}$ eV. We use the Gibbs2 code^45–47^ to calculate the thermodynamic properties of the heterostructures within the framework of the quasi-harmonic Debye model (see Supplementary Information).

**Figure 1:**
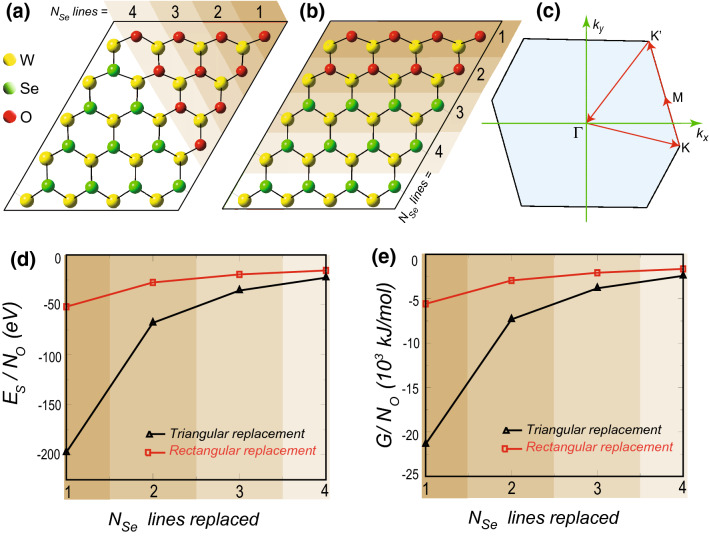
$4 \times 5$ super cell of 2D monolayer of $\hbox {WSe}_{2}$ with (**a**) triangular and (**b**) rectangular O substitution, respectively. The different shades, along with the numeric values 1–4 show the lines of Se atoms replaced by O upon gradual oxidation. (**c**) The distorted hexagonal first Brillouin zone of $4 \times 5$ super cell with high-symmetric points. (**d**) The stabilization energy ($E_S$) and (**e**) the molar Gibbs free energy (*G*) at room temperature (298 K) and 0 GPa pressure, normalized to the number of substituted O atoms ($N_O$) as a function of the number of Se lines replaced in triangular (triangle) and rectangular (square) fashion. The shades in (**d**) and (**e**) correspond to that of (**a**) and (**b**).

## Results and discussion

We begin by taking a $4 \times 5$ super cell of a 2D monolayer of $\hbox {WSe}_{2}$ and gradually replace the lines of Se atoms with lines of O atoms, step-by-step, in a triangular and in a rectangular fashion as shown in Fig. 1a,b, respectively. Due to the asymmetry in super cell geometry, the hexagonal first Brillouin zone gets distorted, as can be seen in Fig. 1c. For ab-initio energetics study, we first optimize the structures and then calculate the stabilization energies of this tungsten selenide-oxide in-plane heterostructures (see Table S1 of Supplementary Information). The stabilization energy is defined as, $E_S = E_{Total} - \sum _{i}N_iE_i$, where $E_S$ is the stabilization energy, $E_{Total}$ is the total energy of the system at room temperature (298 K) and 0 GPa pressure, as obtained from the Gibbs2 code^45–47^. $N_i$ and $E_i$ are the number and the energy of a single atom of the *i*-th species ($i=$ W, Se, O), respectively. We have seen an increase in stabilization energy upon gradual layer-by-layer oxidation of few-layer TMDs in our previous study^36^. Here, the gradual in-plane oxidation of monolayer $\hbox {WSe}_{2}$ too provides higher stabilization. Since the overall stabilization energy increases with the number of O atom replacement, to compare the stability of the triangular and rectangular oxide islands, we present the stabilization energy normalized to the number of substituted O atoms as a function of the number of Se lines replaced in Fig. 1d. It clearly shows that the stability of triangular oxide islands is higher than that of the rectangular ones. The stabilization energy plots for both the geometrical shapes approach towards a saturation value, which is the characteristic stabilization energy of monolayer $\hbox {WO}_{2}$, that is when O atoms replace all the Se atoms. We have not explicitly included the contribution of zero point energy (ZPE) originating from the atomic vibrational modes in calculating $E_S$ due to expensive phonon frequency computations.

However, we consider the Debye model in quasi-harmonic approximation as implemented in the Gibbs2 code^45–47^ to implicitly account for the effect of zero point energy (ZPE) and the vibrational contribution to the enthalpy and entropy (see Supplementary Information). Moreover, we theoretically probe the thermal effects on the TMD-based oxide heterostructures’ stability since, in experiments, the layer-by-layer oxidation is temperature-controlled^35^. The molar Gibbs Free Energy at room temperature (298 K) and 0 GPa pressure, derived from the model (see Fig. S1–S8 in Supplementary Information), is normalized to the number of O atoms as a function of the number of Se lines replaced, as shown in Fig. 1e (see also the Table S1 of Supplementary Information). We observe that the thermodynamic stability trend, as depicted in Fig. 1e, is identical to the structural stability due to $E_S$, as shown in Fig. 1d. Hence, the thermodynamic calculations clearly demonstrate that the triangular phases of the heterostructures are more stable than the rectangular phases.

**Figure 2:**
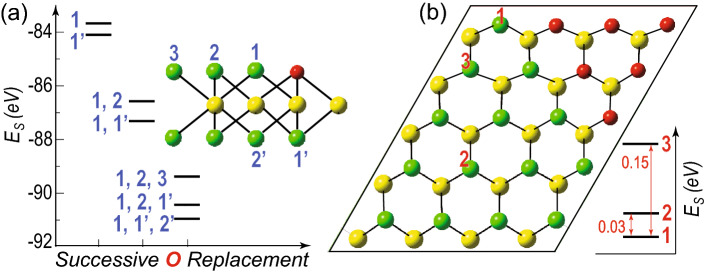
(**a**) $2 \times 2$ super cell of a 2D monolayer of $\hbox {WSe}_{2}$ with one Se atom of the top surface replaced by the O atom. The numeric values 1, 2, 3 ( 1’, 2’) show the location of further O replacement in the top (bottom) surface. The adjoining plot shows the stabilization energy of the system (horizontal line) with successive O replacement. The associated numeric value corresponding to each symbol shows the position of the O replacements in the system. (**b**) $4 \times 5$ super cell of a 2D monolayer of $\hbox {WSe}_{2}$ with triangular O substitution ($N_O$ = 12). The numeric values 1, 2, 3 show the next O substitution location in the system. The adjoining plot shows the relative stabilization energy of the system (horizontal line) with O substitution. The numeric value associated with each symbol shows the location of the O substitution in the system. Both the stabilization energy schemes in (**a**) and (**b**) highlight O atom diffusion’s most energetically favorable position in these TMD systems.

Having discussed the stability of the triangular oxide islands, we investigate the energetics of the Se substitution by O and the trend for the clusterization of the oxygen atoms into triangular patches at zero temperature. This is related to the short- and long-range ordering of the oxygen atoms within the two layers of chalcogen atoms. We consider a $2 \times 2$ supercell of a 2D monolayer of $\hbox {WSe}_{2}$ and replace one of the Se atoms at the top surface by O atom initially. Now, we investigate the most probable positions of further O replacement, as depicted by the numeric in Fig. 2a. We observe that the Se atom at immediately adjacent bottom surface (position 1’) to the initial O atom is energetically more favorable to be replaced than that in the top surface (position 1). The same trend continues with successive Se replacements (see Fig. 2a). This shows the nucleation for oxidation prefers the Se replacement in the top and bottom surfaces simultaneously rather than replacing Se atoms step-by-step in the entire top surface. This gives a clear indication of how the oxide islands initially nucleate in the pristine TMD layer.

Furthermore, to explain the preferential triangular self-formation of the oxide islands, we consider a $4 \times 5$ super cell of the selenide-oxide heterostructure with pre-formed triangular oxide islands and investigate the preference of the next Se atom replacement, as depicted by the numeric in Fig. 2b. The corresponding relative stabilization energy plot shows that the Se atom (position 1) adjacent to the pre-formed oxide triangle is energetically more favorable to be replaced by the O atom. Therefore, the oxide island preferentially grows in a compact triangular fashion rather than dispersed in random positions (2 and 3). This theoretically explains the triangular growth morphology of the 2D TMD oxide heterostructures, as seen in experiments^35^.

**Figure 3:**
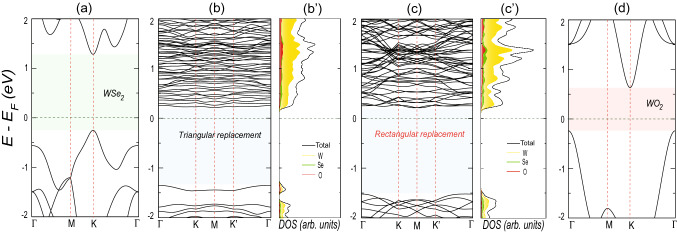
(**a**) Energy dispersion of pristine 2D monolayer of $\hbox {WSe}_{2}$. The energy dispersions of in-plane selenide-oxide heterostructures with triangular (as in Fig. 1a) and rectangular (as in Fig.1b) oxide islands are shown in (**b**) and (**c**), along with their density of states in (**b**’) and (**c**’), respectively. The projected density of states from W, Se, and O atoms are also shown in different shades. The energy dispersion of monolayer $\hbox {WO}_{2}$ is shown in (**d**). In all the energy dispersion plots, the horizontal (vertical) dashed lines show the location of Fermi energy (high-symmetric points). Note that, in case of (**a**) pristine $\hbox {WSe}_{2}$ and (**d**) $\hbox {WO}_{2}$, we consider single unit cell and regular hexagonal first Brillouin zone.

We further investigate the electronic properties of this in-plane heterostructures. It is well known that monolayer TMDs are direct band gap semiconductors, as shown in Fig. 3a. This results in a characteristic peak in photoluminescence spectra^35,48^. However, it has been observed that upon oxidation, this peak disappears^35^. In Fig. 3b, we present the energy dispersion of 2D $\hbox {WSe}_{2}$ monolayer with triangular oxide island, as shown in Fig. 1a. It shows an indirect band gap semiconducting behavior, explaining the photoluminescence peak’s disappearance, as observed in the previous experiments. The projected density of states (see Fig. 3b’) shows the hybridization of W, Se, and O orbitals in the entire energy window. The top-most valence band, which arises mainly from the W and O orbitals, gets detached from the bulk valence bands and shows almost dispersion-less behavior. Actually, the triangular oxide islands behave like discrete quantum dots, embedded in the $\hbox {WSe}_{2}$ plane with negligible hopping among them and therefore result in the dispersion-less character of the top-most valence band. On the contrary, the rectangular oxide island as shown in Fig. 1b also shows an indirect band gap semiconducting behavior (see Fig. 3c,c’). However, due to the periodic nature of the quasi-1D rectangular oxide strip, no dispersion-less band arises near Fermi energy, unlike its triangular counterpart. Complete oxidation of the monolayer $\hbox {WSe}_{2}$ leads to an indirect band gap semiconductor (see Fig. 3d) and consequent disappearance of photoluminescence peak. The energy dispersions for the systems with progressive triangular and rectangular oxidation are presented in Fig. S9–S14 in Supplementary Information. As can be seen, the systems with different sizes of triangular oxide islands show a band gap of $\sim$ 1.6–1.8 eV. The consideration of hybrid functionals can produce a more accurate band gap than the GGA-PBE level of calculation that is known to underestimate the same^49^. Therefore, it is expected that these systems with triangular oxide islands will show optical absorption in the visible spectrum with possible light-harvesting applications.

## Conclusion

To summarize, we have investigated the reason behind the preferential triangular growth morphology of oxide islands on monolayer $\hbox {WSe}_{2}$ that can be generalized for other 2D pristine TMDs. Our ab-initio energetics and thermodynamic study show that the origin of these intriguing triangular oxides in TMD systems is due to the preferential O substitution at selenium zigzag edges. This lends credence to experimental results^35,50^ and can be extended to most 2D TMDs. Moreover, the TMD based oxide heterostructures can have potential use in various electronic and optoelectronic device applications.

## Supplementary Information

Supplementary Information
