# Supplementary material for: Ab-initio investigation of preferential triangular self-formation of oxide heterostructures of monolayer WSe2
Source: Sci Rep. 2020 Dec 10;10:21737. doi: 10.1038/s41598-020-78812-2 (PMC7729869; doi:10.1038/s41598-020-78812-2)
Supplement: Supplementary file 1 — Supplementary Information [file 41598_2020_78812_MOESM1_ESM.pdf]

Electronic Supplementary Information

Ab Initio Investigation of Preferential Triangular Self-formation of Oxide Heterostructures of Monolayer WSe_2

Soumya Ranjan Das[1], Katsunori Wakabayashi[2,3], Kazuhito Tsukagoshi[3] and Sudipta Dutta*[1]

[1]*Department of Physics, Indian Institute of Science Education and Research (IISER) Tirupati, Tirupati- 517507, Andhra Pradesh, India.*

[2]*Department of Nanotechnology for Sustainable Energy, School of Science and Technology, Kwansei Gakuin University, Gakuen 2-1, Sanda 669-1337, Hyogo, Japan.*

[3]*WPI Center for Materials Nanoarchitectonics (WPI-MANA), National Institute for Materials Science (NIMS), Tsukuba 305-0044, Japan.*

AUTHOR INFORMATION

Corresponding Author:

*Sudipta Dutta. *Email:* sdutta@iisertirupati.ac.in

Theoretical Methodology for Thermodynamic Stability Calculation

In our paper, we determine the thermodynamic stability of Transition-Metal Dichalcogenide (TMD) based oxide heterostructures (see Figs. S1(a) to S8(a)) by employing the quasi-harmonic Debye model as implemented in the Gibbs2 code[1-3]. In this section, we briefly describe our theoretical methodology. Our motivation is to include the thermal effects in the thermodynamic phase stability calculations with a minimal set of *ab initio* data, using an intuitive yet completely general non-empirical model.

We use density functional theory (DFT) as implemented in the Vienna *ab initio* simulation package (VASP)[4] for structural relaxation of the heterostructures, as described in the manuscript. After obtaining the equilibrium geometry, we select a grid of volumes encompassing the equilibrium volume by changing the lattice parameter and find the total energy of the solid (*E*) as a function of the molecular volume (*V*). With the static calculations, we obtain the *E(V)* curve (see Figs. S1(b) to S8(b)).

The Gibbs2 code[1-3] is then used to minimize the non-equilibrium Gibbs free energy per mole[5],

$$G^*(\mathbf{x}_{opt}(V); p, T) = E(\mathbf{x}_{opt}(V)) + pV + A_{vib}(\mathbf{x}_{opt}(V); T) \quad (1)$$

where, $\mathbf{x}_{opt}$ is the optimized configuration vector that includes the geometric information of the system, $E(\mathbf{x}_{opt}(V))$ is the total energy of the system, pV corresponds to the constant hydrostatic pressure, and the last term, A_{vib} is the vibrational Helmholtz free energy[1]. The quasi-harmonic Debye model is used to express the vibrational contribution as[5,6]

$$A_{vib}(\Theta; T) = nkT[\frac{9}{8}\frac{\Theta}{T} + 3ln(1 - e^{-\frac{\Theta}{T}}) - D(\Theta/T)] \quad (2)$$

where $D(y)$ is the Debye integral defined as

$$D(y) = \frac{3}{y^3}\int_0^y \frac{x^3}{e^x - 1}dx \quad (3)$$

Θ is the Debye temperature, n is the number of atoms per formula unit and k is the Boltzmann constant.

We use the quasi-harmonic approximation (QHA) to include the effects of anharmonicity only for realistic simulation of physical systems. In QHA, Θ depends only on the volume. Hence, the static non-equilibrium Gibbs energy per mole is then minimized with respect to the volume to obtain the equation of state (EOS) and the chemical potential, equivalent to the molar Gibbs function (*G)* that governs the thermodynamic stability. We have computed *G* at room temperature (see Fig. 1(e) in the manuscript) and also at other temperatures to explore the thermal effects in the stability of our heterostructure systems (see Figs. S1(c) to S8(c)). To reduce the minimization process of $G^*(V;p,T)$, we fit an approximate analytical function of volume. The fitting of the *E(V)* data gives us an analytical form of the Debye temperature, which can be used to obtain the vibrational Helmholtz free energy and consequently the non-equilibrium Gibbs function polynomial for any selection of temperature and pressure. We have used the Birch-Murnaghan family of EOS to fit the *ab initio E(V)* data in our calculations.[7–9]

Our theoretical methodology, as implemented in the Gibbs2 code[1-3], is quite robust and has been used earlier to study the thermodynamic properties of a wide variety of crystal structures, especially two-dimensional materials like graphene[10] and TMD based systems[11,12].

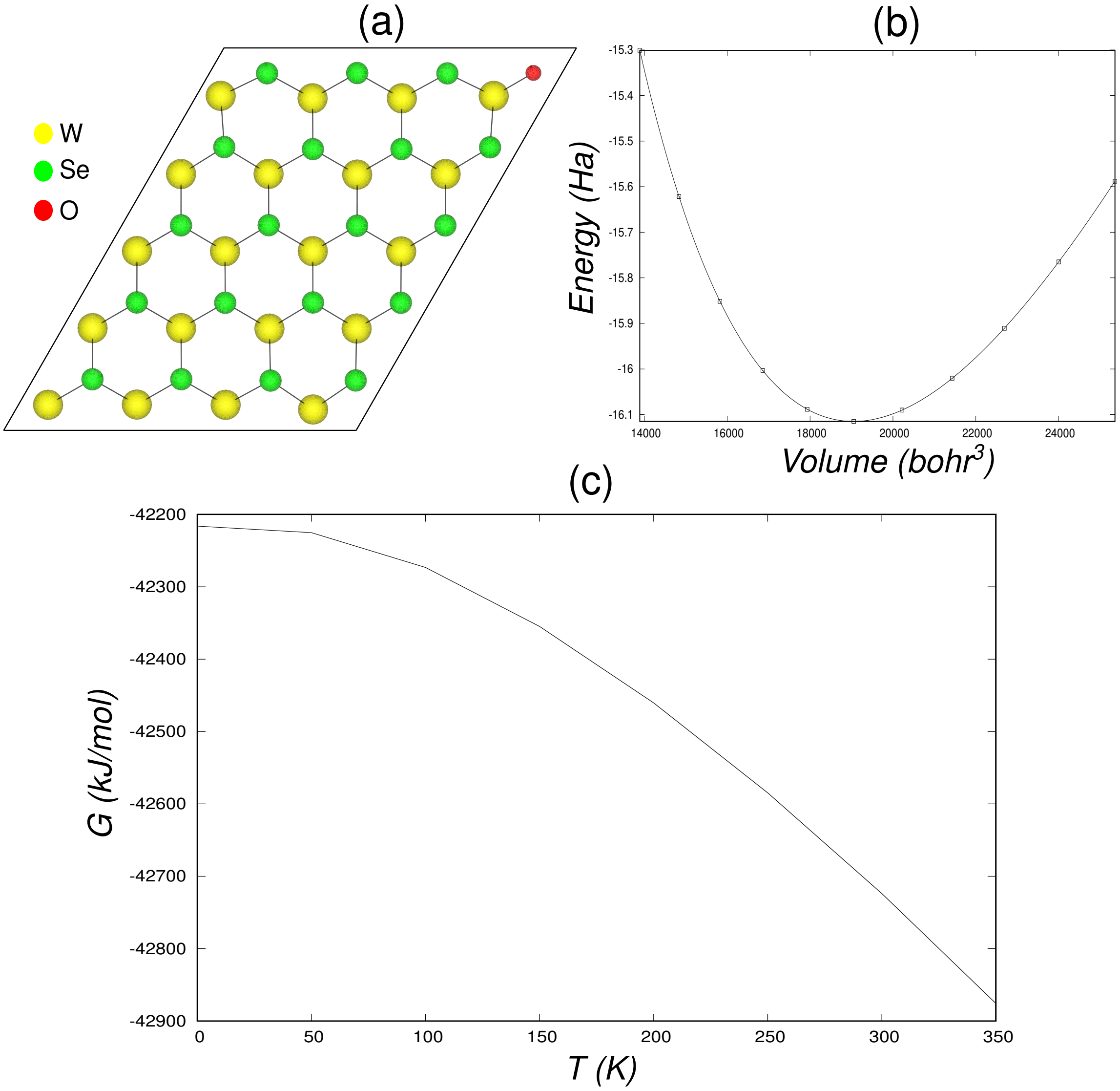

Fig. S1 (a) 4 x 5 super cell of 2D monolayer of WSe_2 with triangular O substitution (N_O = 2). (b) and (c) denote the E(V) curve along with the fitted polynomial in the Birch-Murnaghan strain and the Gibbs free energy per mole as a function of temperature and 0 GPa pressure of the WSe_2 oxide heterostructure system, respectively. The equilibrium lattice constant of the system is 24.92 Bohr radius. Note that, for a pristine WSe_2 unit cell, the equilibrium lattice constant is 6.24 Bohr (3.3 Å). Since, here we are considering a 4 x 5 super cell, the unit cell lattice constant along the x-axis has to be multiplied by 4.

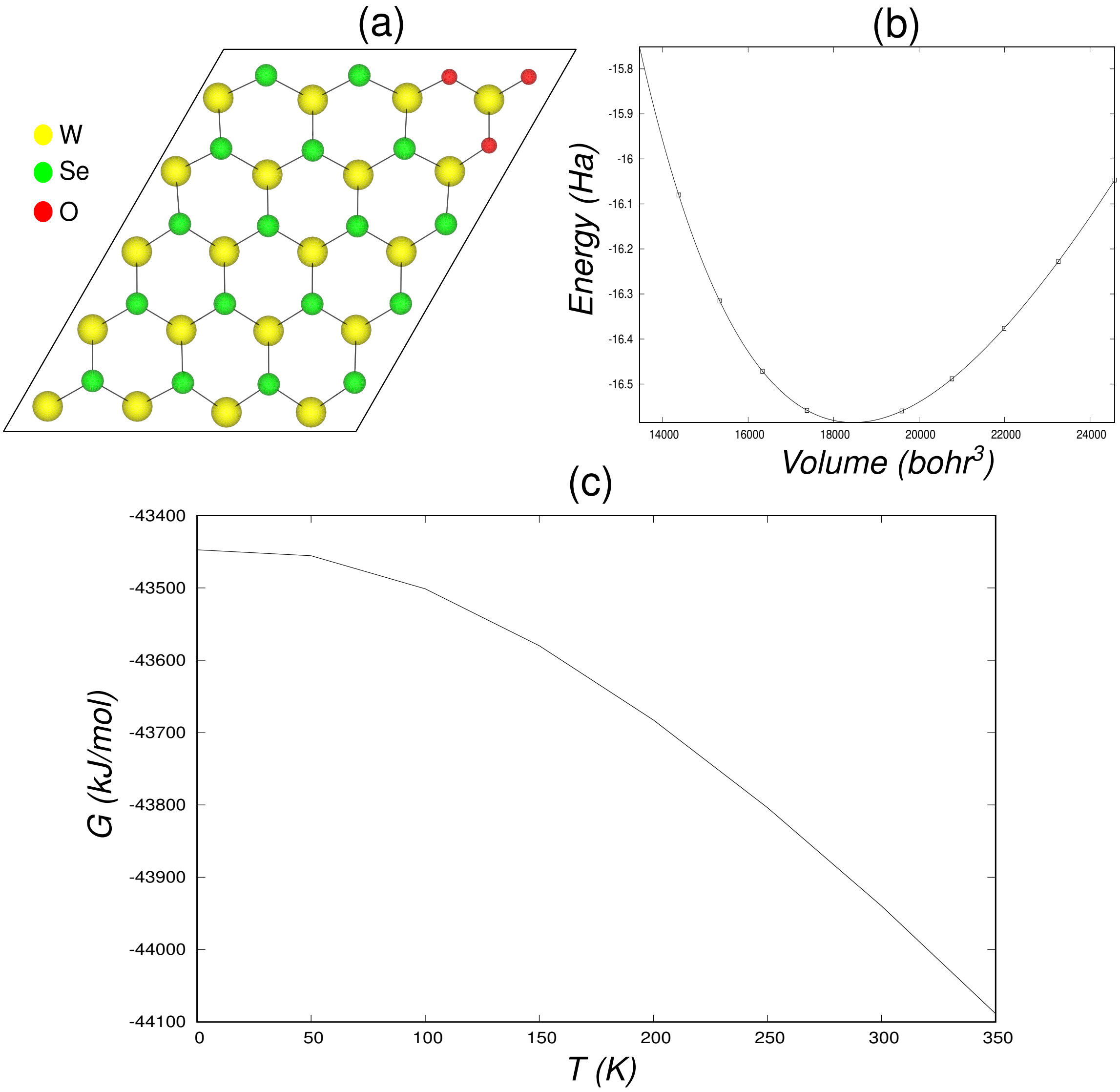

Fig. S2 (a) 4 x 5 super cell of 2D monolayer of WSe_2 with triangular O substitution ($N_O = 6$). (b) and (c) denote the E(V) curve along with the fitted polynomial in the Birch-Murnaghan strain and the Gibbs free energy per mole as a function of temperature and 0 GPa pressure of the WSe_2 oxide heterostructure system, respectively. The equilibrium lattice constant of the system is 24.54 Bohr radius.

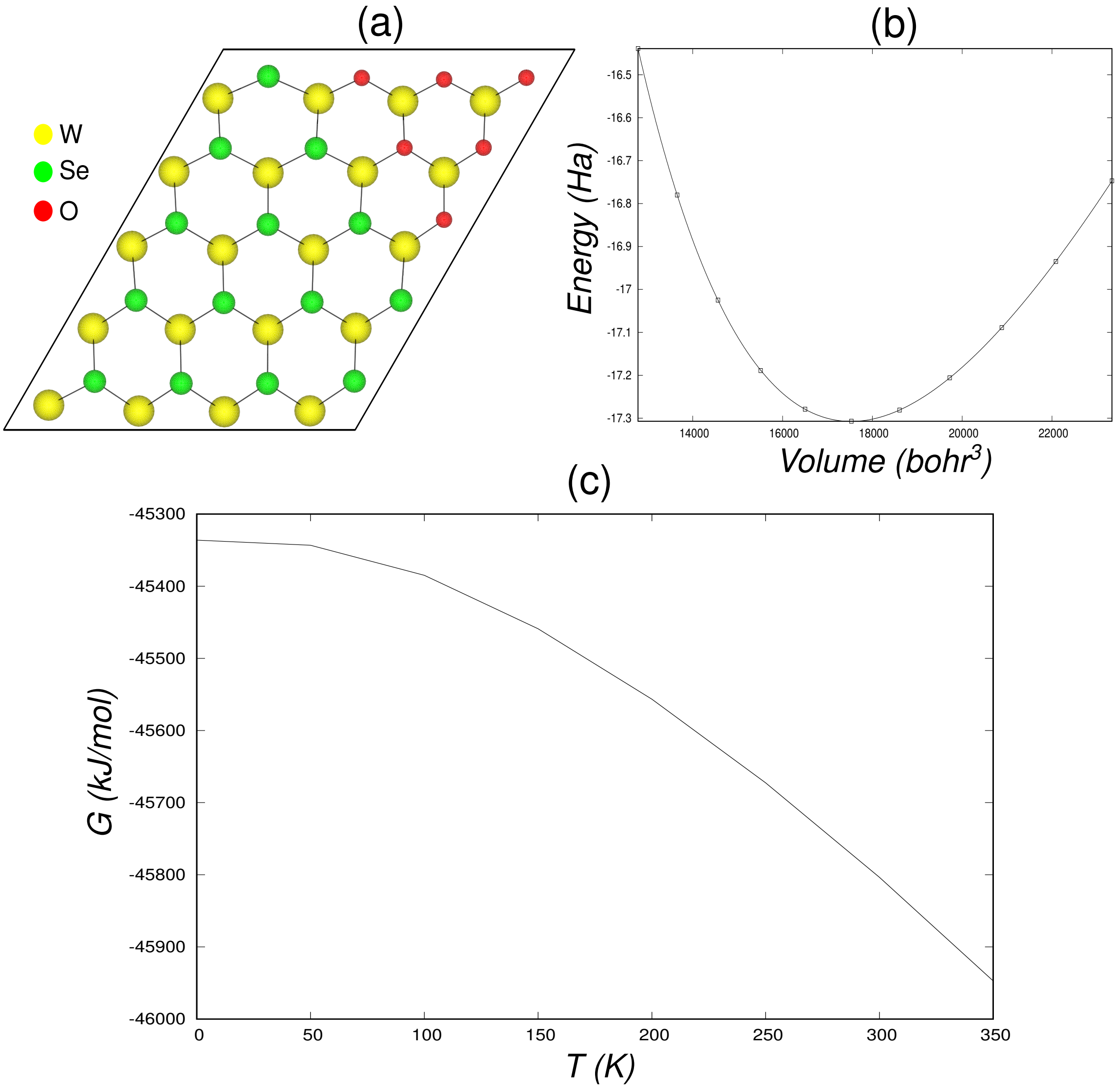

Fig. S3 (a) 4 x 5 super cell of 2D monolayer of WSe_2 with triangular O substitution (N_O = 12). (b) and (c) denote the *E(V)* curve along with the fitted polynomial in the Birch-Murnaghan strain and the Gibbs free energy per mole as a function of temperature and 0 GPa pressure of the WSe_2 oxide heterostructure system, respectively. The equilibrium lattice constant of the system is 23.99 Bohr radius.

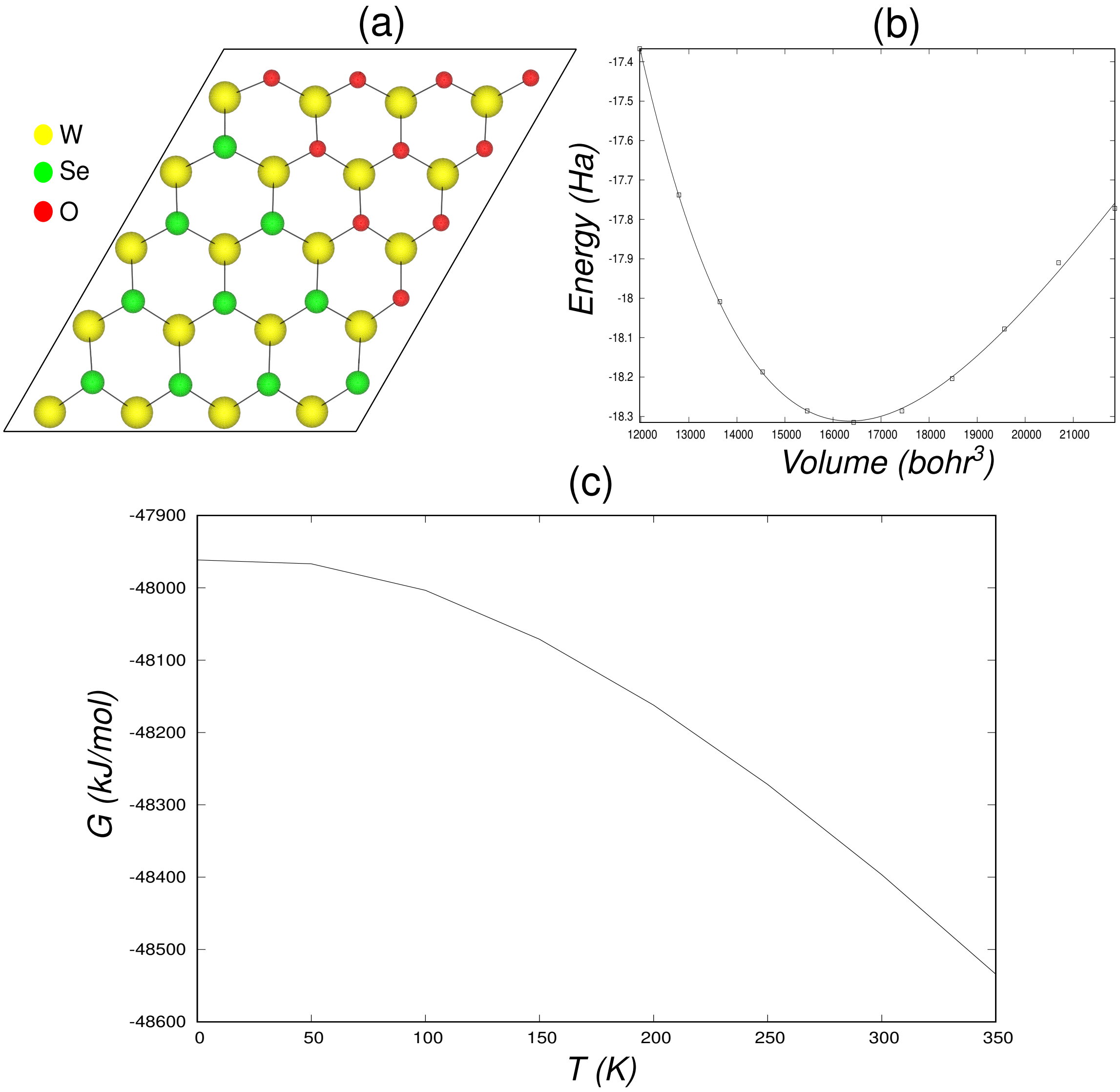

Fig. S4 (a) 4 x 5 super cell of 2D monolayer of WSe_2 with triangular O substitution ($N_O = 20$). (b) and (c) denote the E(V) curve along with the fitted polynomial in the Birch-Murnaghan strain and the Gibbs free energy per mole as a function of temperature and 0 GPa pressure of the WSe_2 oxide heterostructure system, respectively. The equilibrium lattice constant of the system is 23.19 Bohr radius.

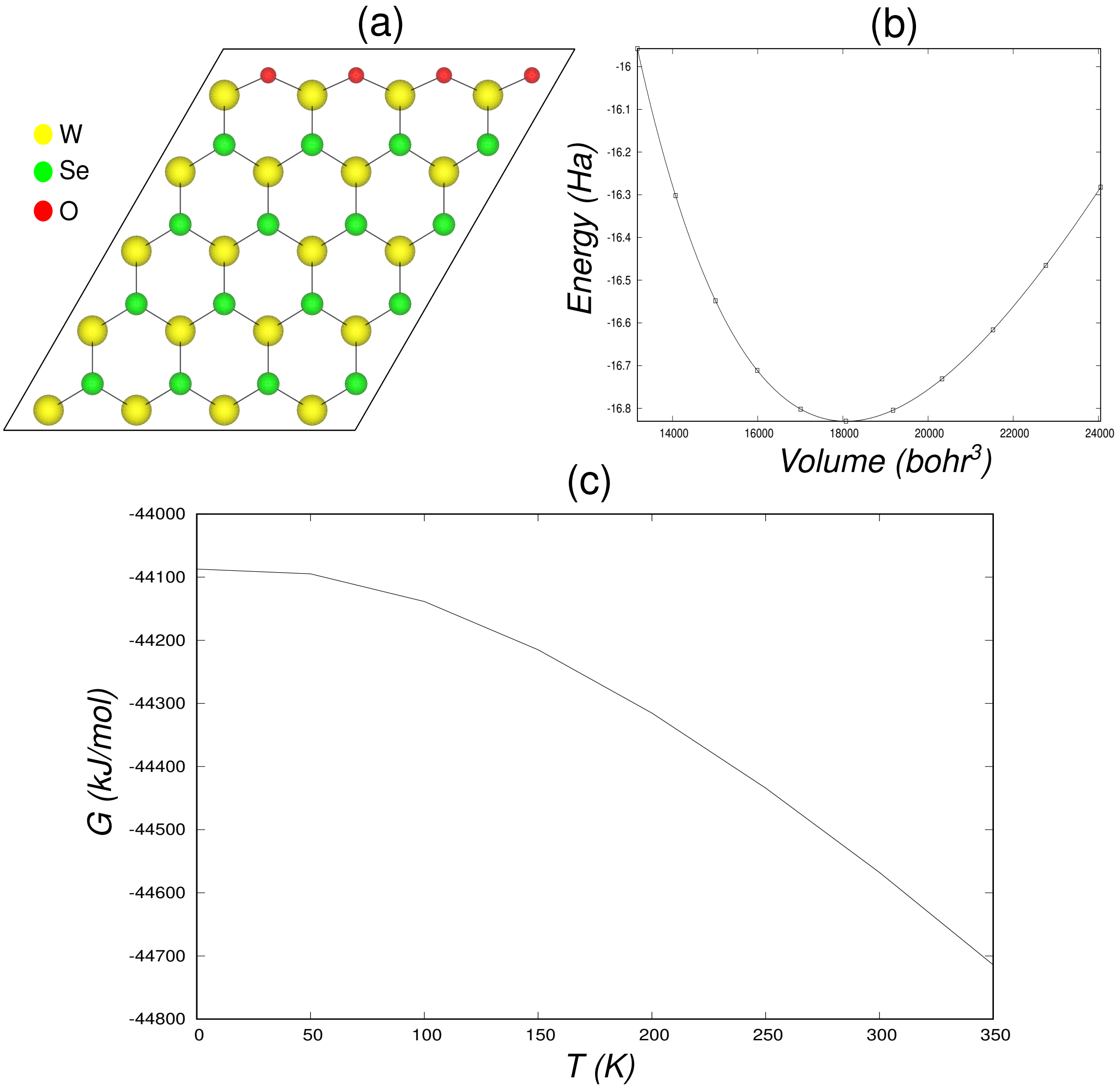

Fig. S5 (a) 4 x 5 super cell of 2D monolayer of WSe_2 with rectangular O substitution ($N_O = 8$). (b) and (c) denote the E(V) curve along with the fitted polynomial in the Birch-Murnaghan strain and the Gibbs free energy per mole as a function of temperature and 0 GPa pressure of the WSe_2 oxide heterostructure system, respectively. The equilibrium lattice constant of the system is 24.38 Bohr radius.

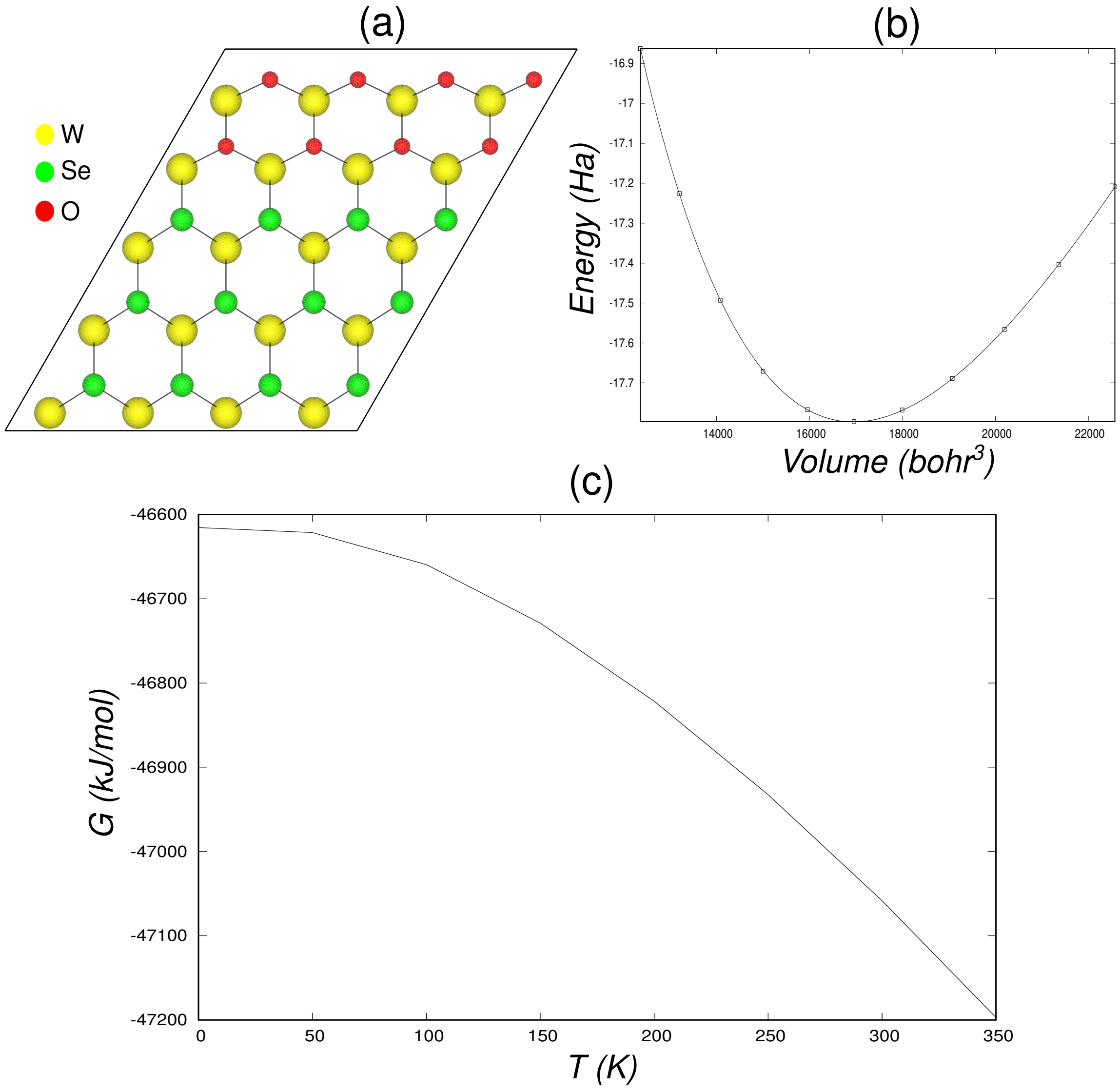

Fig. S6 (a) 4 x 5 super cell of 2D monolayer of WSe_2 with rectangular O substitution (N_O = 16). (b) and (c) denote the E(V) curve along with the fitted polynomial in the Birch-Murnaghan strain and the Gibbs free energy per mole as a function of temperature and 0 GPa pressure of the WSe_2 oxide heterostructure system, respectively. The equilibrium lattice constant of the system is 23.65 Bohr radius.

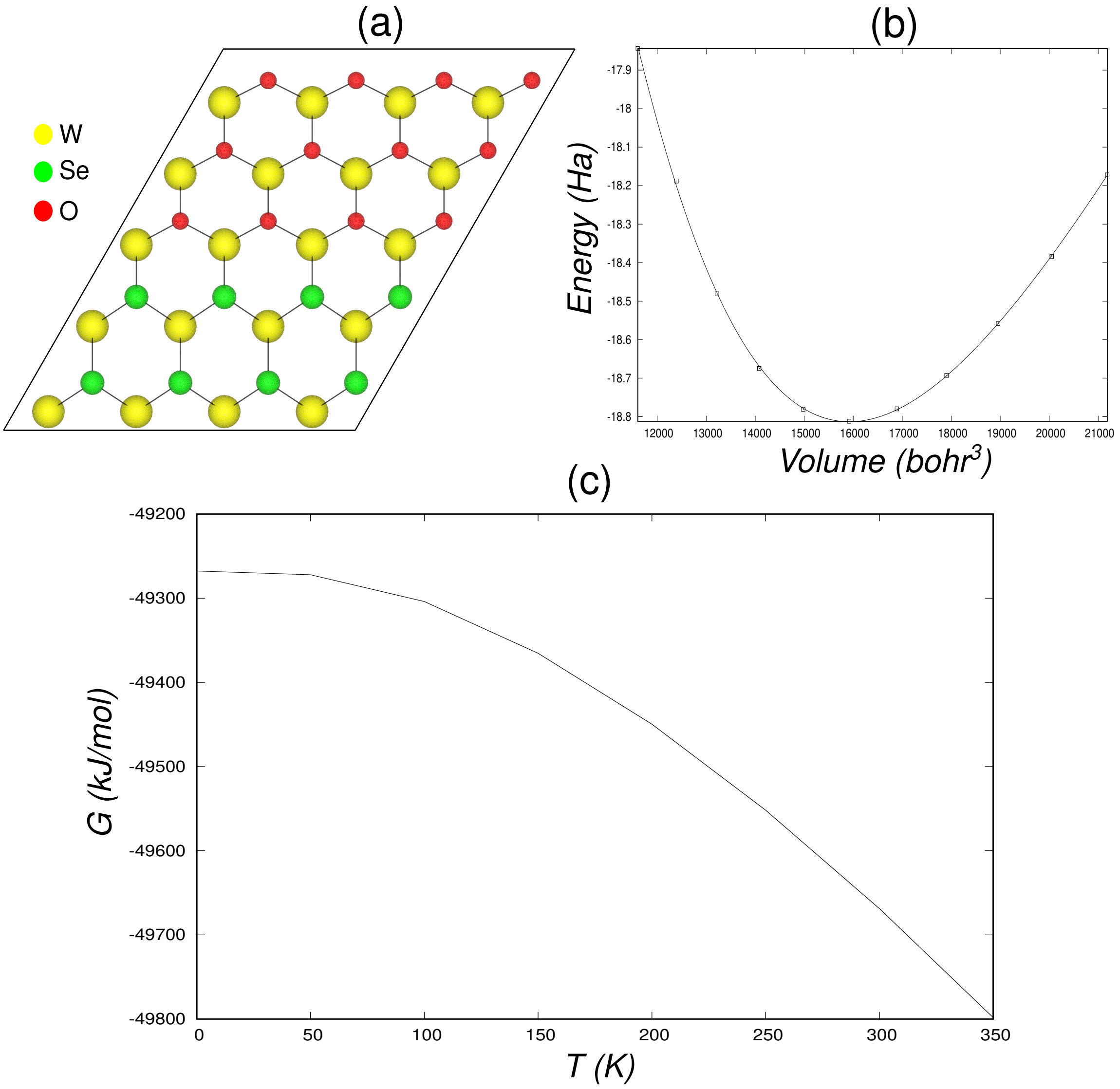

Fig. S7 (a) 4 x 5 super cell of 2D monolayer of WSe_2 with rectangular O substitution (N_O = 24). (b) and (c) denote the E(V) curve along with the fitted polynomial in the Birch-Murnaghan strain and the Gibbs free energy per mole as a function of temperature and 0 GPa pressure of the WSe_2 oxide heterostructure system, respectively. The equilibrium lattice constant of the system is 22.94 Bohr radius.

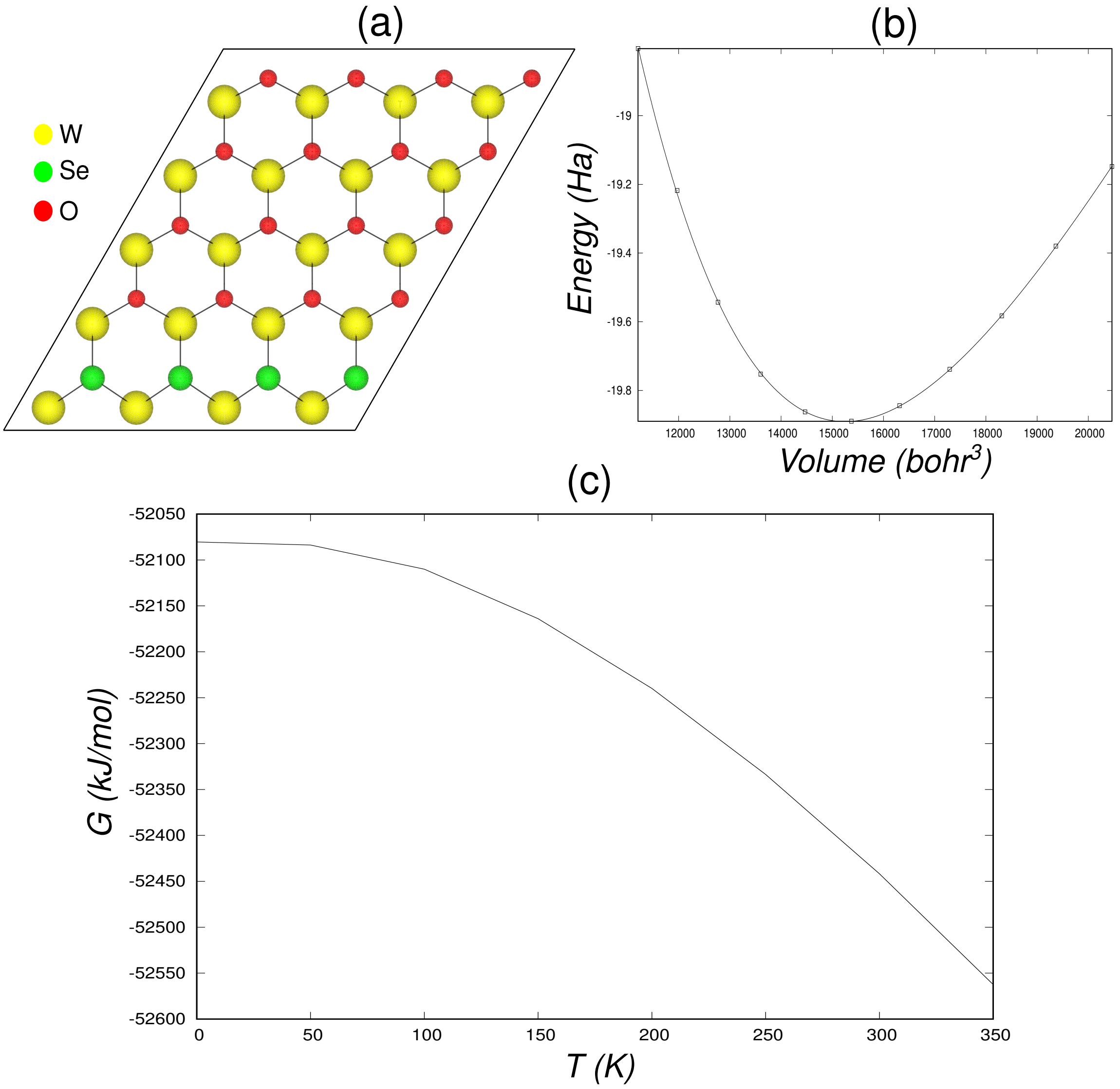

Fig. S8 (a) 4 x 5 super cell of 2D monolayer of WSe_2 with rectangular O substitution (N_O = 32). (b) and (c) denote the E(V) curve along with the fitted polynomial in the Birch-Murnaghan strain and the Gibbs free energy per mole as a function of temperature and 0 GPa pressure of the WSe_2 oxide heterostructure system, respectively. The equilibrium lattice constant of the system is 22.23 Bohr radius.

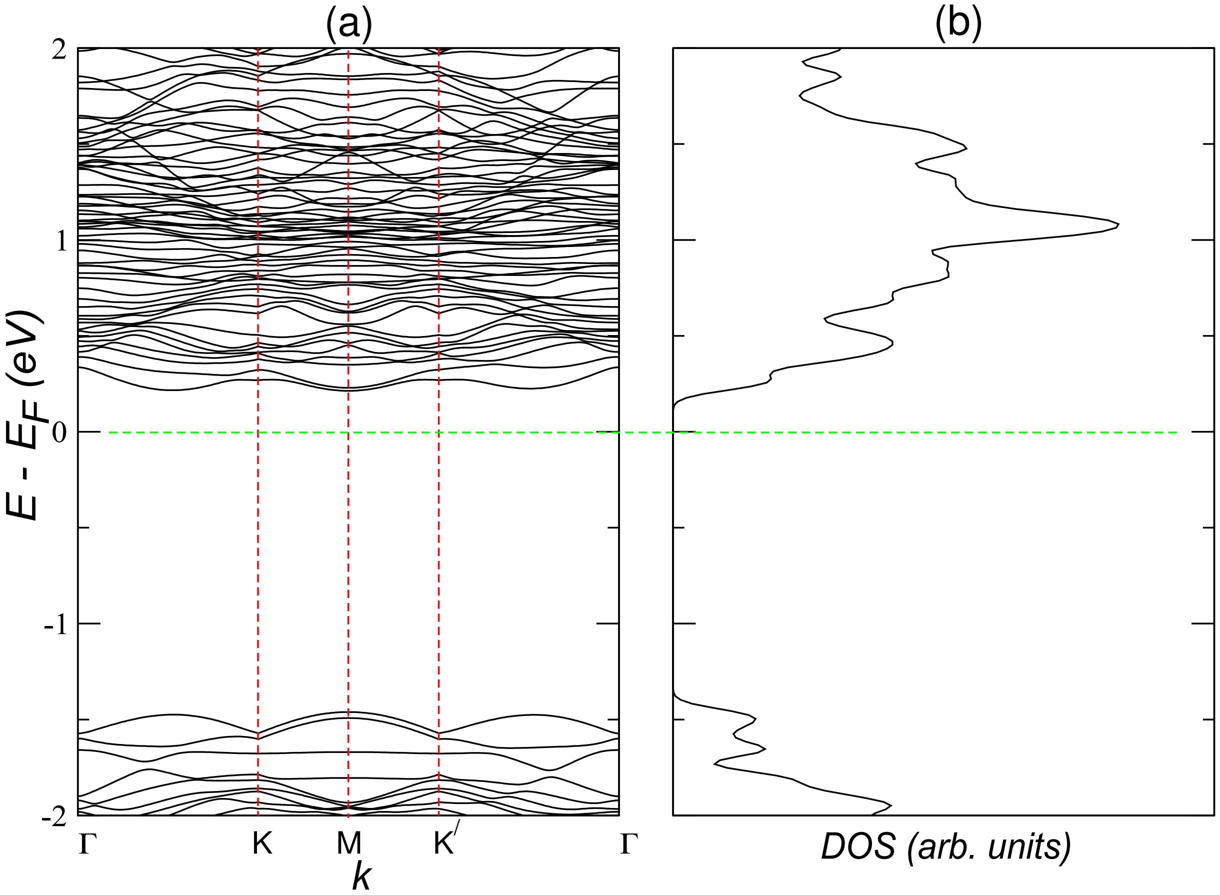

Fig. S9 (a) and (b) denote the band structure and the density of states (DOS) of 4 x 5 super cell of 2D monolayer of WSe_2 with triangular O substitution ($N_O = 2$), respectively (see Fig. S1(a)). The horizontal (vertical) dashed lines show the location of the Fermi energy (high-symmetric points).

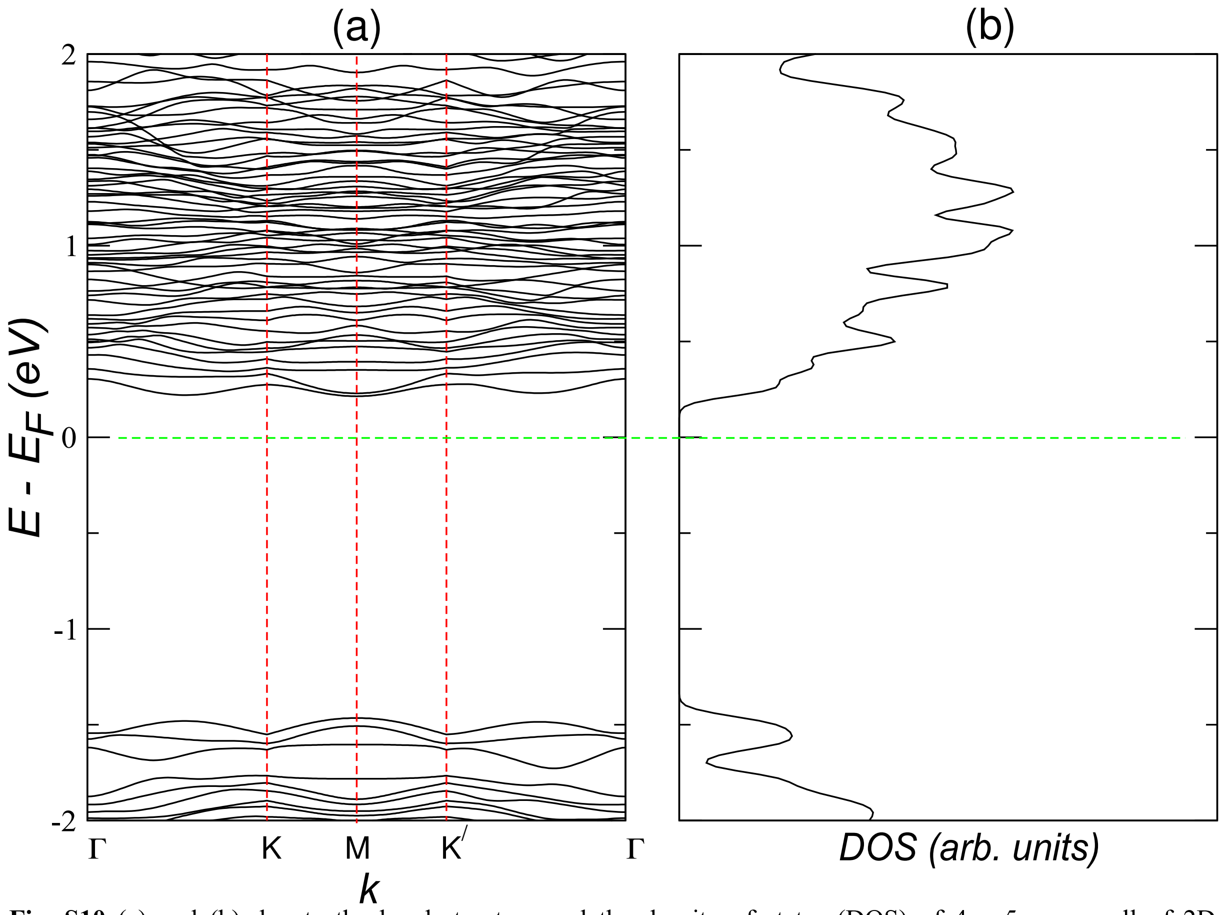

Fig. S10 (a) and (b) denote the band structure and the density of states (DOS) of 4 x 5 super cell of 2D monolayer of WSe_2 with triangular O substitution (N_O = 6), respectively (see Fig. S2(a)). The horizontal (vertical) dashed lines show the location of the Fermi energy (high-symmetric points).

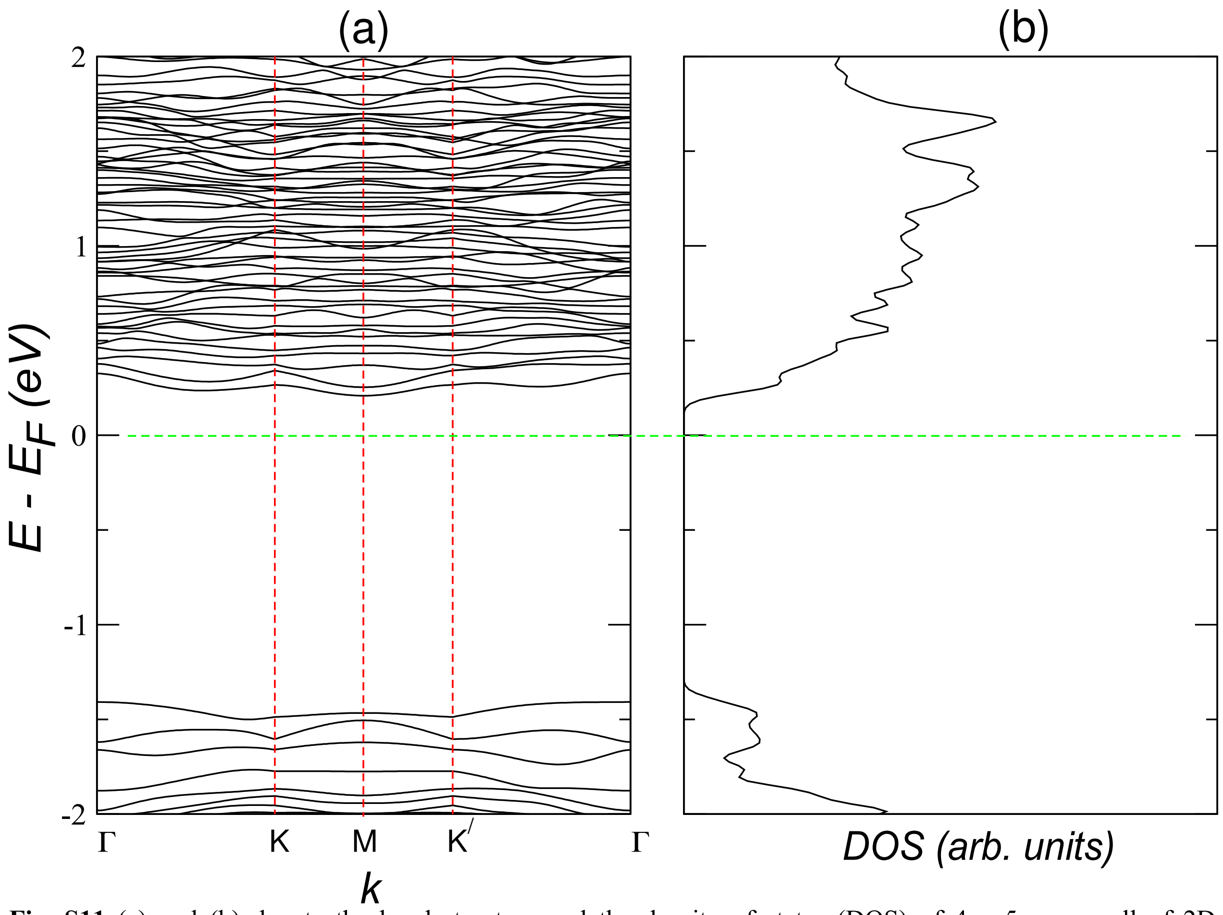

Fig. S11 (a) and (b) denote the band structure and the density of states (DOS) of 4 x 5 super cell of 2D monolayer of WSe_2 with triangular O substitution (N_O = 12), respectively (see Fig. S3(a)). The horizontal (vertical) dashed lines show the location of the Fermi energy (high-symmetric points).

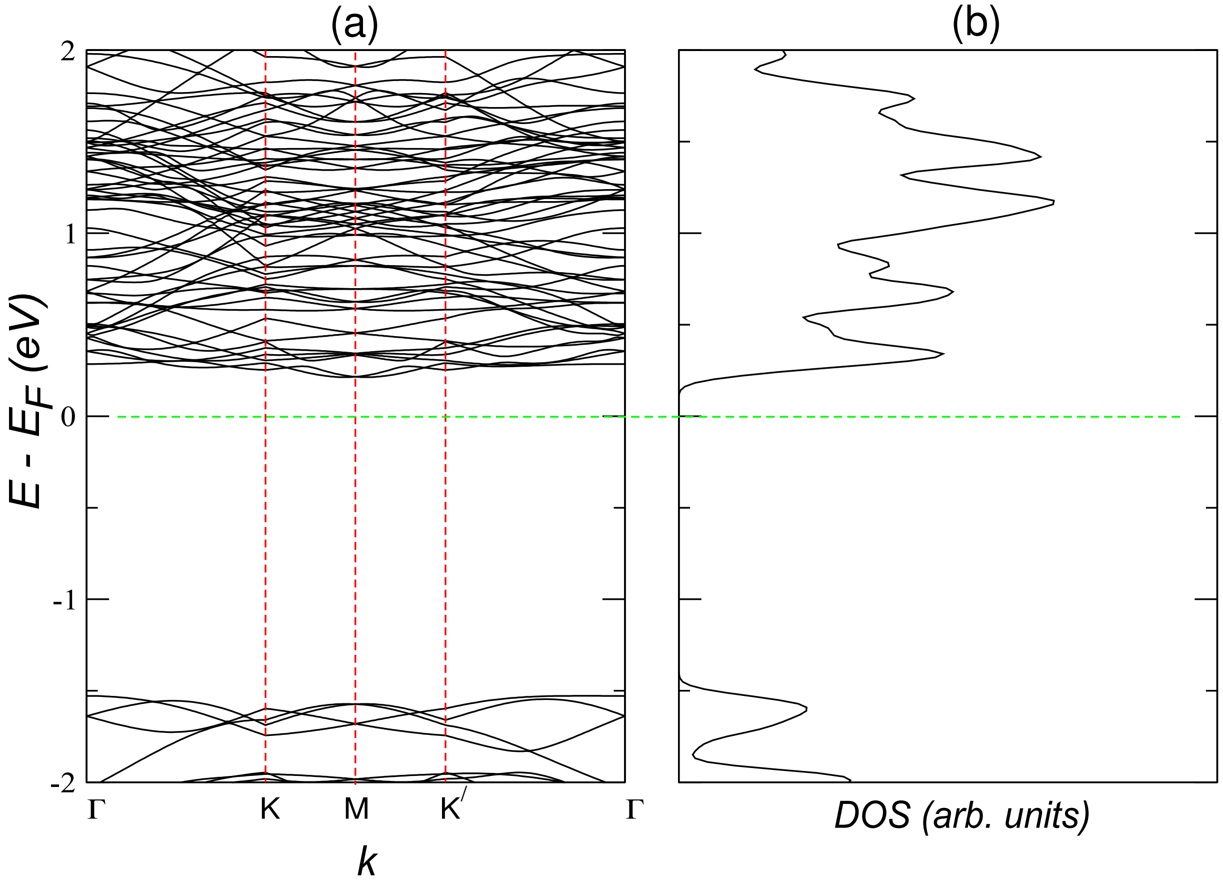

Fig. S12 (a) and (b) denote the band structure and the density of states (DOS) of 4 x 5 super cell of 2D monolayer of WSe_2 with rectangular O substitution (N_O = 8), respectively (see Fig. S5(a)). The horizontal (vertical) dashed lines show the location of the Fermi energy (high-symmetric points).

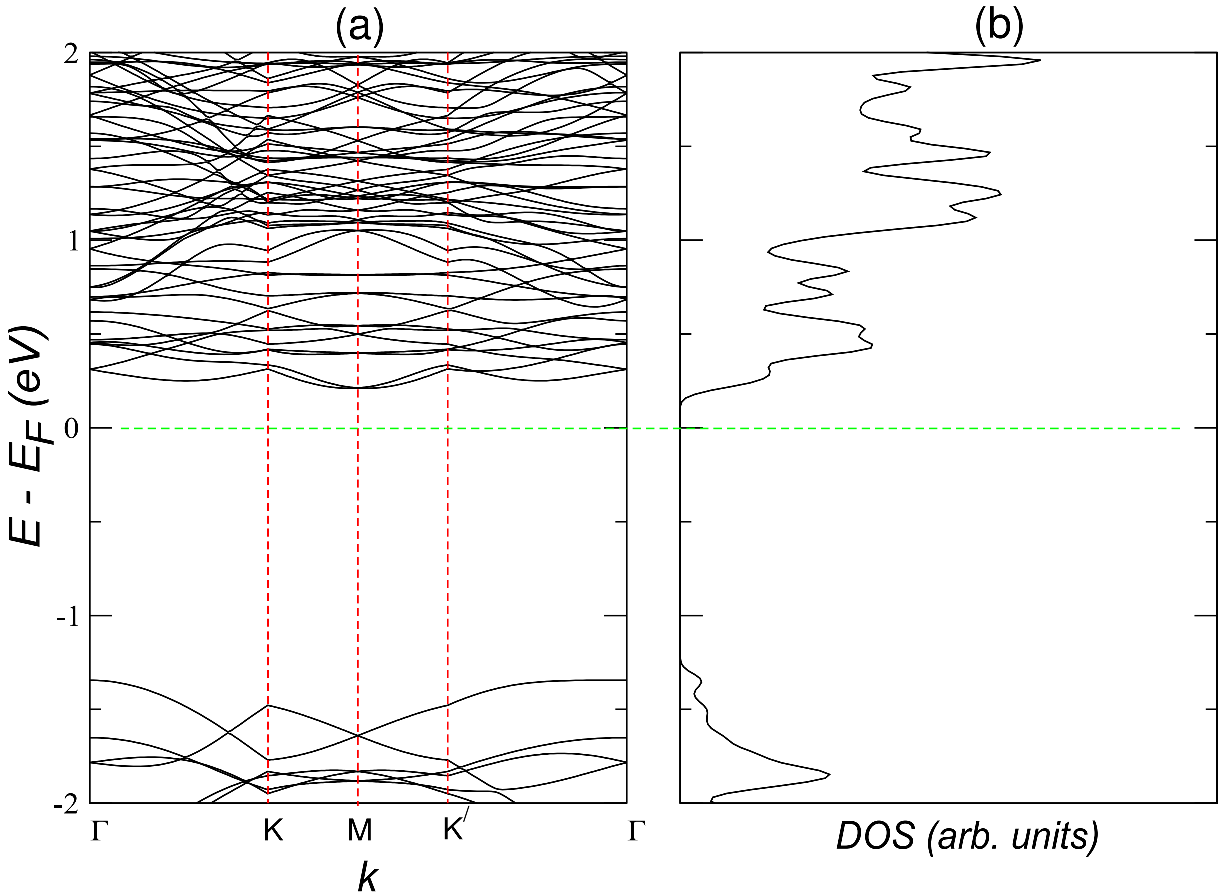

Fig. S13 (a) and (b) denote the band structure and the density of states (DOS) of 4 x 5 super cell of 2D monolayer of WSe_2 with rectangular O substitution (N_O = 24), respectively (see Fig. S7(a)). The horizontal (vertical) dashed lines show the location of the Fermi energy (high-symmetric points).

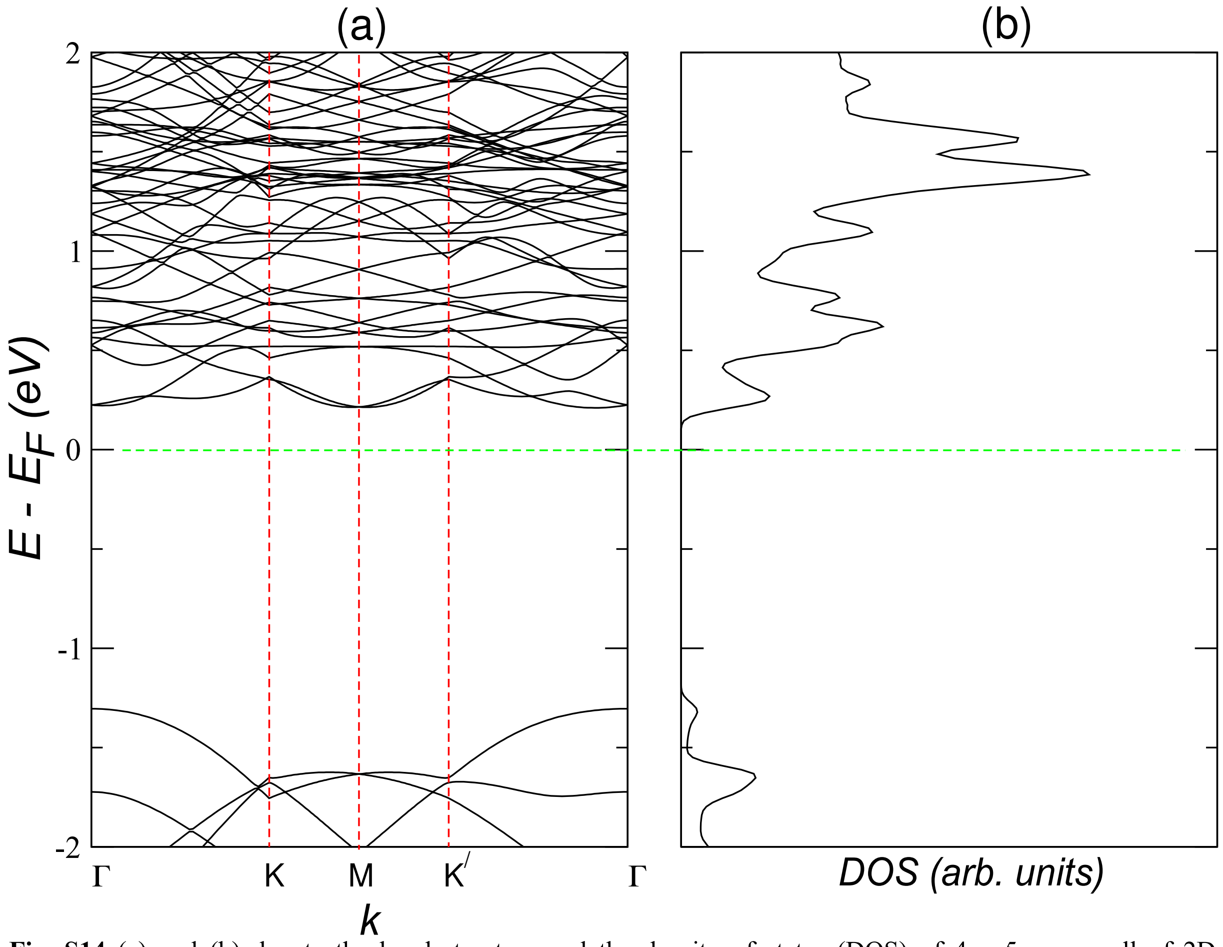

Fig. S14 (a) and (b) denote the band structure and the density of states (DOS) of 4 x 5 super cell of 2D monolayer of WSe_2 with rectangular O substitution (N_O = 32), respectively (see Fig. S8(a)). The horizontal (vertical) dashed lines show the location of the Fermi energy (high-symmetric points).

Table S1 The stabilization energy (E_S) and the Gibbs free energy (G) per oxygen atom substituted (N_O) at room temperature (298 K) and 0 GPa pressure of 4 x 5 super cell of 2D monolayer of WSe_2 with step-by-step replacement of Se with O atoms in a triangular or rectangular fashion, for the structures depicted in Figs. S1(a) to Fig. S8(a).

Structure	E_S/N_O ***(eV)***	G/N_O ***(10^3 kJ/mol)***
Triangular O substitution (N_O = 2)	-197.514	-21.359
Triangular O substitution (N_O = 6)	-67.97	-7.322
Triangular O substitution (N_O = 12)	-35.629	-3.817
Triangular O substitution (N_O = 20)	-22.743	-2.420
Rectangular O substitution (N_O = 8)	-51.816	-5.570
Rectangular O substitution (N_O = 16)	-27.555	-2.940
Rectangular O substitution (N_O = 24)	-19.524	-2.069
Rectangular O substitution (N_O = 32)	-15.561	-1.639

References

1. Blanco, M. A., Francisco, E. & Luaña, V. GIBBS: isothermal-isobaric thermodynamics of solids from energy curves using a quasi-harmonic Debye model. *Comput. Phys. Commun.* **158**, 57–72 (2004).
2. Otero-de-la-Roza, A. & Luaña, V. Gibbs2: A new version of the quasi-harmonic model code. I. Robust treatment of the static data. *Comput. Phys. Commun.* **182**, 1708–1720 (2011).
3. Otero-de-la-Roza, A., Abbasi-Pérez, D. & Luaña, V. Gibbs2: A new version of the quasiharmonic model code. II. Models for solid-state thermodynamics, features and implementation. *Comput. Phys. Commun.* **182**, 2232–2248 (2011).
4. Kresse, G. & Furthmüller, J. Efficient iterative schemes for *ab initio* total-energy calculations using a plane-wave basis set. *Phys. Rev. B* **54**, 11169–11186 (1996).
5. Maradudin, A. A., Montroll, E. W., Weiss, G. H. & Ipatova, I. P. *Theory of lattice dynamics in the harmonic approximation*. (Acad. Press, 1971).
6. Francisco, E., Blanco, M. A. & Sanjurjo, G. Atomistic simulation of SrF_2 polymorphs. *Phys. Rev. B* **63**, 094107 (2001).
7. Murnaghan, F. D. The Compressibility of Media under Extreme Pressures. *Proc. Natl. Acad. Sci. U.S.A.* **30**, 244–247 (1944).
8. Birch, F. Finite Elastic Strain of Cubic Crystals. *Phys. Rev.* **71**, 809–824 (1947).
9. Birch, F. Finite strain isotherm and velocities for single-crystal and polycrystalline NaCl at high pressures and 300°K. *J. Geophys. Res.* **83**, 1257 (1978).
10. Kheyri, A. & Nourbakhsh, Z. First principle calculations of thermodynamic properties of pure graphene sheet and graphene sheets with Si, Ge, Fe, and Co impurities. *Chinese Phys. B* **25**, 093102 (2016).
11. Yuan, J. *et al.* First-principles study of the phonon vibrational spectra and thermal properties of hexagonal MoS_2. *Solid State Sci.* **40**, 1–6 (2015).
12. Zhang, H.-Y., Xi, F., Zeng, Z.-Y., Chen, X.-R. & Cai, L.-C. First-Principles Predictions of Phase Transition and Mechanical Properties of Tungsten Diboride under Pressure. *J. Phys. Chem. C* **121**, 7397–7403 (2017).
